# Nanomicellar Formulation of Clotrimazole Improves Its Antitumor Action toward Human Breast Cancer Cells

**DOI:** 10.1371/journal.pone.0130555

**Published:** 2015-06-22

**Authors:** Mariah C. Marcondes, Anne C. S. Fernandes, Ivaldo Itabaiana, Rodrigo O. M. A. de Souza, Mauro Sola-Penna, Patricia Zancan

**Affiliations:** 1 Laboratório de Oncobiologia Molecular (LabOMol), Departamento de Biotecnologia Farmacêutica (BioTecFar), Faculdade de Farmácia, Universidade Federal do Rio de Janeiro, Rio de Janeiro, RJ, Brasil; 2 Instituto de Microbiologia Paulo de Góes, Universidade Federal do Rio de Janeiro, Rio de Janeiro, RJ, Brasil; 3 Laboratório de Biocatálise e Síntese Orgânica, Instituto de Química, Universidade Federal do Rio de Janeiro, Rio de Janeiro, RJ, Brasil; 4 Laboratório de Enzimologia e Controle do Metabolismo (LabECoM), Departamento de Biotecnologia Farmacêutica (BioTecFar), Faculdade de Farmácia, Universidade Federal do Rio de Janeiro, Rio de Janeiro, RJ, Brasil; University of Nebraska Medical Center, UNITED STATES

## Abstract

**Background:**

Although demonstrated as a selective anticancer drug, the clinical use of clotrimazole (CTZ) is limited due to its low solubility in hydrophilic fluids. Thus, we prepared a water-soluble nanomicellar formulation of CTZ (_n_CTZ) and tested on the human breast cancer cell line MCF-7 biology.

**Methodology/Principal Findings:**

CTZ was nanoencapsulated in tween 80 micelles, which generated nanomicelles of, approximately, 17 nm of diameter. MCF-7 cells were treated with _n_CTZ and unencapsulated DMSO-solubilized drug (_s_CTZ) was used for comparison. After treatment, the cells were evaluated in terms of metabolism, proliferation, survival and structure. We found that _n_CTZ was more efficient than _s_CTZ at inhibiting glycolytic and other cytosolic and mitochondrial enzymes. Moreover, this increased activity was also observed for lactate production, intracellular ATP content, ROS production and antioxidant potential. As a consequence, _n_CTZ-treated MCF-7 cells displayed alterations to the plasma membrane, mitochondria and the nucleus. Finally, _n_CTZ induced both apoptosis and necrosis in MCF-7 cells.

**Conclusions/Significance:**

MCF-7 cells are more sensible to _n_CTZ than to _s_CTZ. This was especially evident on regard to antioxidant potential, which is an important cell defense against drugs that affect cell metabolism. Moreover, this water-soluble formulation of CTZ strengths its potential use as an anticancer medicine.

## Introduction

Cancer chemotherapy continues to suffer from the same problem: undesirable and toxic side effects [1–3]. Most of these side effects are due to the non-specificity of the drugs currently used. Therefore, the search for specific drugs with minor effects on non-tumor cells has received increasing attention [1,4–6]. Several therapies based on monoclonal antibodies have emerged to specifically deliver drugs and radiochemicals to cancer cells but, to the best of our knowledge, these therapies still present undesirable side effects due to the antibodies immunoreactivity [7–9]. Many of the pharmaceuticals used for cancer chemotherapy are intended to arrest cell division and hinder proliferation and migration. However, it is largely accepted that among the major hallmarks of cancer cells that can be targeted by chemical intervention, the unique cancer metabolic profile is always a good choice [10,11]. Cancer cells present an accelerated glycolytic rate, which, even in a normal oxygen supply, is directed to the lactic fermentative route [12,13]. At the same time, mitochondria are constantly metabolizing metabolites other than pyruvate—especially glutamine—that are largely used as carbon sources for the biosynthesis of lipids and amino acids primarily for the construction of membranes and proteins, respectively [5,12,14–16]. This aerobic glycolytic preference of cancer cells is named the Warburg effect for its first description by Otto Warburg in 1956 [17].

Drugs directly targeting glycolytic enzymes and thus addressing the Warburg effect, such as 3-bromopyruvate (inhibitor of hexokinase) [18–20] and clotrimazole (phosphofructokinase inhibitor) [21–25], are specific for cancer cells with minor effects on normal cells. Initially promoted as the solution for cancer chemotherapy, many of these drugs failed due undesirable side effects [26–30] and, in the case of clotrimazole and many others, the low solubility in hydrophilic media [31–33]. Clotrimazole (CTZ) is an antifungal azole derivative, which has been used as an antitumoral agent due to its properties to inhibit cell proliferation [34] by inhibiting glycolysis [35]. CTZ is described to directly inhibit the major regulatory glycolytic enzyme, phosphofructokinase [23,24], which is an important mechanism for its effects on cancer biology. Recently, we have shown that CTZ also directly inhibit phosphatidylinositol-3-kinase (PI3K) as a key mechanism for the antitumoral effects of this drug [36].

Nanotechnology-based drug formulations, the so-called nanomedicines, have gained attention due to their ability to circumvent many pharmaceutical issues, such as solubility, stability and toxicity [1,37–40]. Among the various targeted and non-targeted nanomedicines and a great variety of techniques developed to address distinct pathologies, nanomicelles have emerged as one of the most effective vehicles for drug delivery [1]. Among nanomicelles, the microemulsions are known for their ease of assembly, stability and ability to carry hydrophobic drugs [41]. Microemulsions are produced by stirring a surfactant in water, which generates nanoscale spherical droplets that form a clear and thermodynamically stable system [42]. In the current work, we used the non-ionic surfactant Tween 80 to produce microemulsions, herein named nanomicelles, carrying CTZ to test their effects on the biology of MCF-7 human breast cancer cells. Our results show that CTZ-containing nanomicelles are not only a good carrier for the drug but that they are also more efficient than the soluble drug. Therefore, this preparation might be a candidate for nanomedicine.

## Results

**Fig 1:**
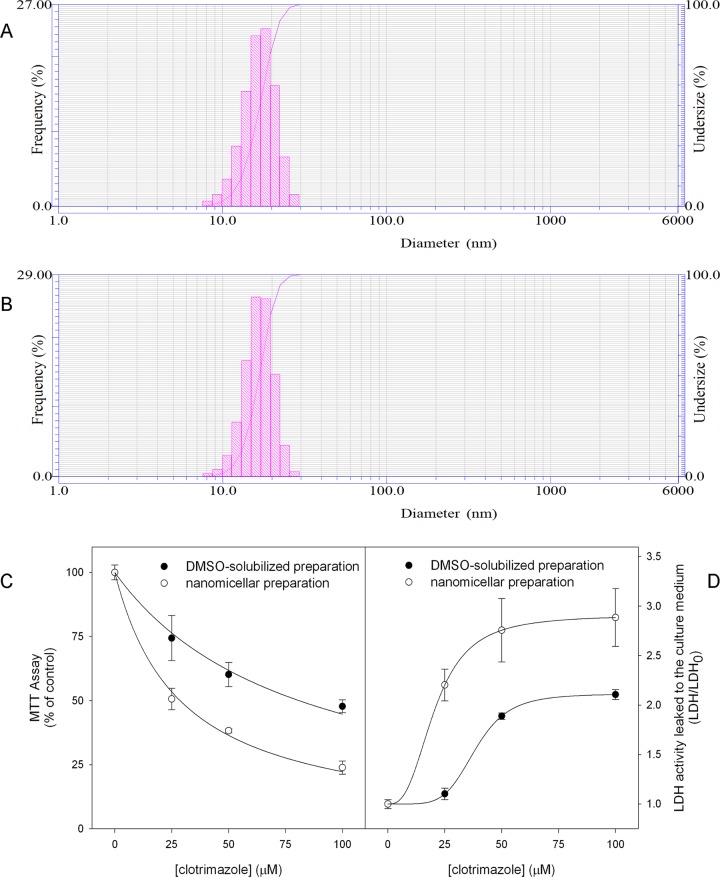
Size characterization and initial effects of CTZ-containing nanomicelles. Nanomicelles were prepared as described in Materials and Methods in the presence or absence of CTZ. The size of the nanomicelles was analyzed by dynamic light scattering as described above. Panel A: nanomicelles prepared in the absence of CTZ. Panel B: CTZ-containing nanomicelles. Panel C: MCF-7 cell viability evaluated by the MTT assay in the presence of soluble CTZ (black circles) or a nanomicellar preparation of the drug (white circles). Panel D: MCF-7 cell viability evaluated by LDH activity that leaked from the cells in the presence of soluble CTZ (black circles) or a nanomicellar preparation of the drug (white circles). Plotted points are the means ± standard error of the mean, for at least 4 independent experiments (n = 4).

With the intention of circumventing the major issue hindering the therapeutic use of CTZ, *i*.*e*. its low solubility, we incorporated CTZ into a nanoscale water-soluble Tween 80 emulsion, as described in Materials and Methods. The physical characterization of these nanoparticles reveals that they have diameters of 16.9 ± 3.4 nm without CTZ and 17.1 ± 3.9 nm for when CTZ is incorporated (Fig 1A and 1B, respectively). That CTZ incorporation does not significantly affect the nanoparticles’ size is indicative of the stability of the formulation. Indeed, CTZ-containing nanoparticles are stable for at least 24 hours at room temperature (data not shown). To evaluate the efficiency of drug delivery, MCF-7 cells were grown to confluence and incubated for 24 hours in the presence of different concentrations of a DMSO-soluble CTZ (_s_CTZ) preparation or a nanomicellar CTZ (_n_CTZ) preparation. After incubation, cell viability was tested with the MTT assay (Fig 1C) and by the leakage of LDH into the culture medium (Fig 1D). Both techniques revealed that _n_CTZ was able to affect MCF-7 viability more efficiently than _s_CTZ, based on the concentrations necessary to significantly affect viability (the half-maximal effect was achieved at approximately 25 μM _n_CTZ and 50 μM _s_CTZ) and the maximum effects for each (_s_CTZ promoted approximately half of the maximum effect observed for _n_CTZ).

**Fig 2:**
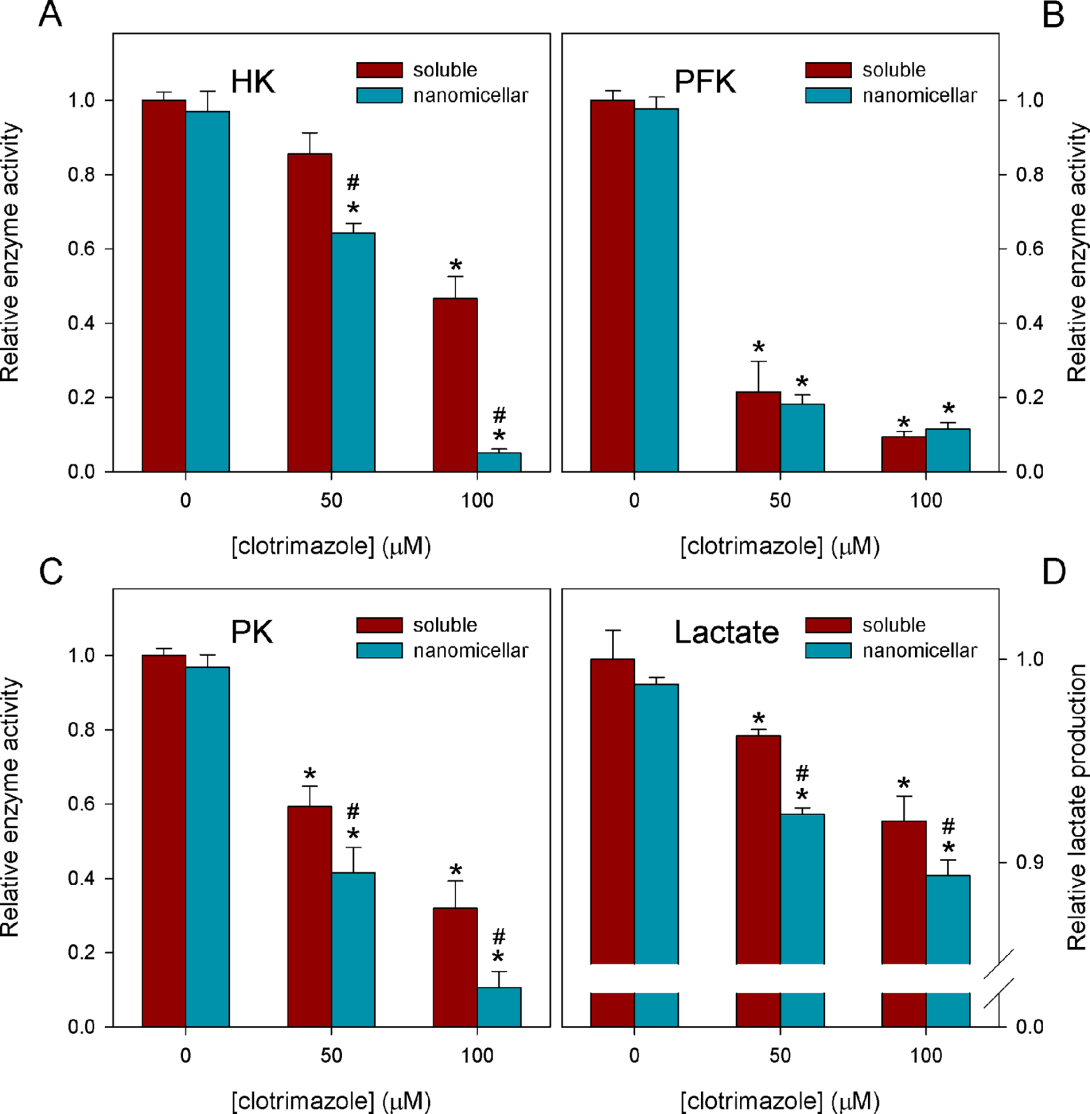
Effects of nanomicellar CTZ on glycolytic parameters. The activities of the glycolytic enzymes HK (panel A), PFK (panel B) and PK (panel C) and lactate production (panel D) were assessed as described in Materials and Methods in the absence and in the presence of 50 or 100 μM of DMSO-solubilized or a nanomicellar preparation of CTZ. In the absence of CTZ the appropriate amount of DMSO (1% vol/vol) or CTZ-free nanomicellar preparation was added as controls. The results for these controls were not different from the control with no addition. Values are the means ± standard error of the mean, for at least 4 independent experiments (n = 4). * P < 0.05 compared to control and # P < 0.05 compared to DMSO-solubilized CTZ (two-tailed ANOVA, Bonferroni’s port-hoc test).

**Fig 3:**
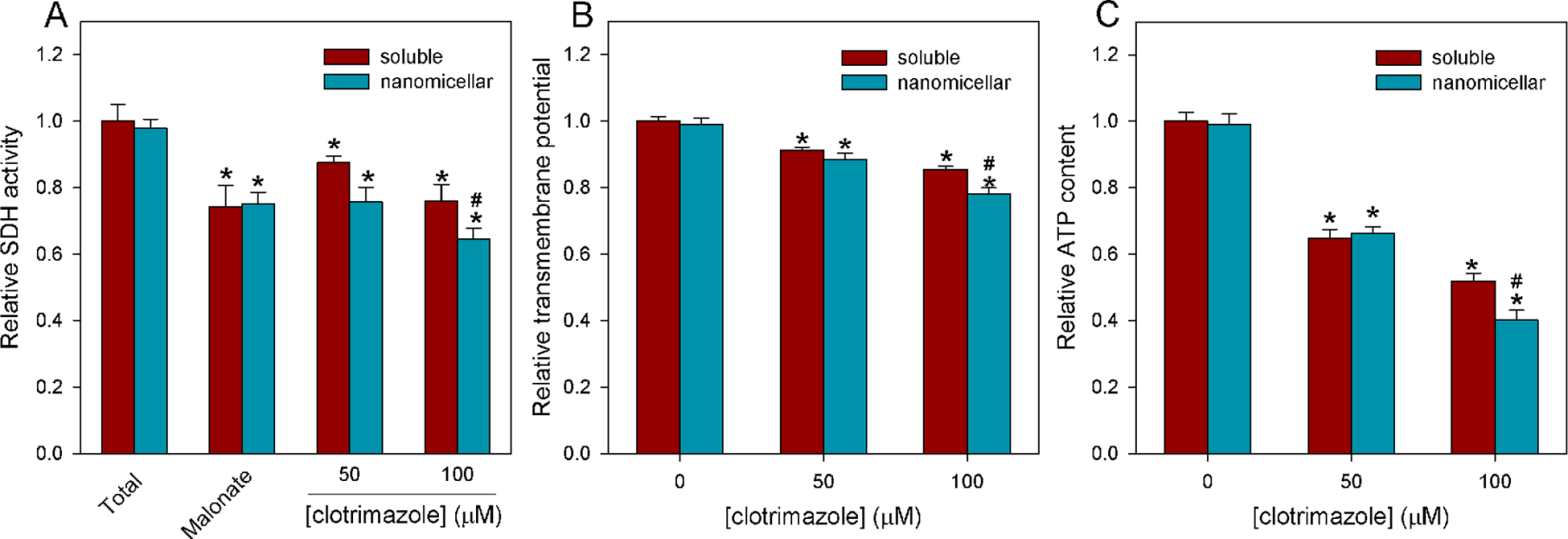
Effects of nanomicellar CTZ on mitochondrial parameters and on intracellular ATP levels. SDH activity (panel A), mitochondrial transmembrane potential (panel B) and intracellular ATP content (panel C) were assessed as described in Materials and Methods in the absence and in the presence of 50 or 100 μM of DMSO-solubilized or a nanomicellar preparation of CTZ. In the absence of CTZ the appropriate amount of DMSO (1% vol/vol) or CTZ-free nanomicellar preparation was added as controls. The results for these controls were not different from the control with no addition. Malonate (12 mM) was used as a control for SDH inhibition. Values are the means ± standard error of the mean, for at least 4 independent experiments (n = 4). * P < 0.05 compared to control and # P < 0.05 compared to DMSO-solubilized CTZ (two-tailed ANOVA, Bonferroni’s port-hoc test).

Because CTZ has been described to exert its antitumor effects by interfering with energy metabolism, we compared the effects of _s_CTZ and _n_CTZ on several metabolic enzymes and markers. For these experiments, we used 50 and 100 μM _s_CTZ or _n_CTZ, as well as the appropriate controls DMSO (soluble) or tween 80 nanomicelles (nanomicellar). It is important to notice that the controls presented were different neither between themselves nor from the control with no addition for all the experiments performed in the present work (data not shown). The major glycolytic regulatory enzymes, hexokinase (HK), phosphofructokinase (PFK) and pyruvate kinase (PK), are inhibited by both _s_CTZ and _n_CTZ (Fig 2). HK and PK are more strongly affected by _n_CTZ than by _s_CTZ (Fig 2A and 2C, for HK and PK, respectively). This higher inhibitory capacity of _n_CTZ compared to _s_CTZ toward HK and PK was observed at both concentrations of the drugs. The effects of _s_CTZ and _n_CTZ on PFK were not significantly different (Fig 2B). However, this enzyme is more sensitive to the inhibitory effects of CTZ than HK and PK. For instance, in the presence of 50 μM _s_CTZ, PFK is inhibited by 80%, whereas HK and PK are only inhibited by 15% and 20%, respectively. Nevertheless, assessing lactate production in MCF-7 cells to measure the glycolytic flux, we observed that overall, glycolysis was more affected by _n_CTZ than by _s_CTZ because _n_CTZ was more effective at decreasing the lactate production rate (Fig 2D). Mitochondrial function was evaluated by measuring succinate dehydrogenase (SDH) activity (Fig 3A) and rhodamine 123 accumulation to assess mitochondrial transmembrane potential (Fig 3B). It is clear that CTZ interferes with mitochondrial functions because the drug inhibits SDH activity and rhodamine 123 accumulation, thus revealing that it diminishes mitochondrial Δψ. Although its effect was more modest toward SDH compared to the glycolytic enzymes, CTZ was similarly comparable to the known inhibitor of this enzyme, malonate (12 mM malonate was used, which has been described to strongly compete with succinate for SDH). _n_CTZ was more efficient than _s_CTZ at 100 μM, but they were similarly effective at 50 μM (Fig 3A). A similar result was observed for mitochondrial transmembrane potential; the difference between _s_CTZ and _n_CTZ was observed only at 100 μM (Fig 3B). Additionally, CTZ diminishes the levels of intracellular ATP, and despite the greater efficiency of _n_CTZ compared to _s_CTZ with regard to the glycolytic parameters (Fig 2), _n_CTZ was only more efficient than _s_CTZ at decreasing ATP levels at 100 μM (Fig 3C). This last result suggests that mitochondrial metabolism supports ATP production in MCF-7 cells exposed to CTZ.

**Fig 4:**
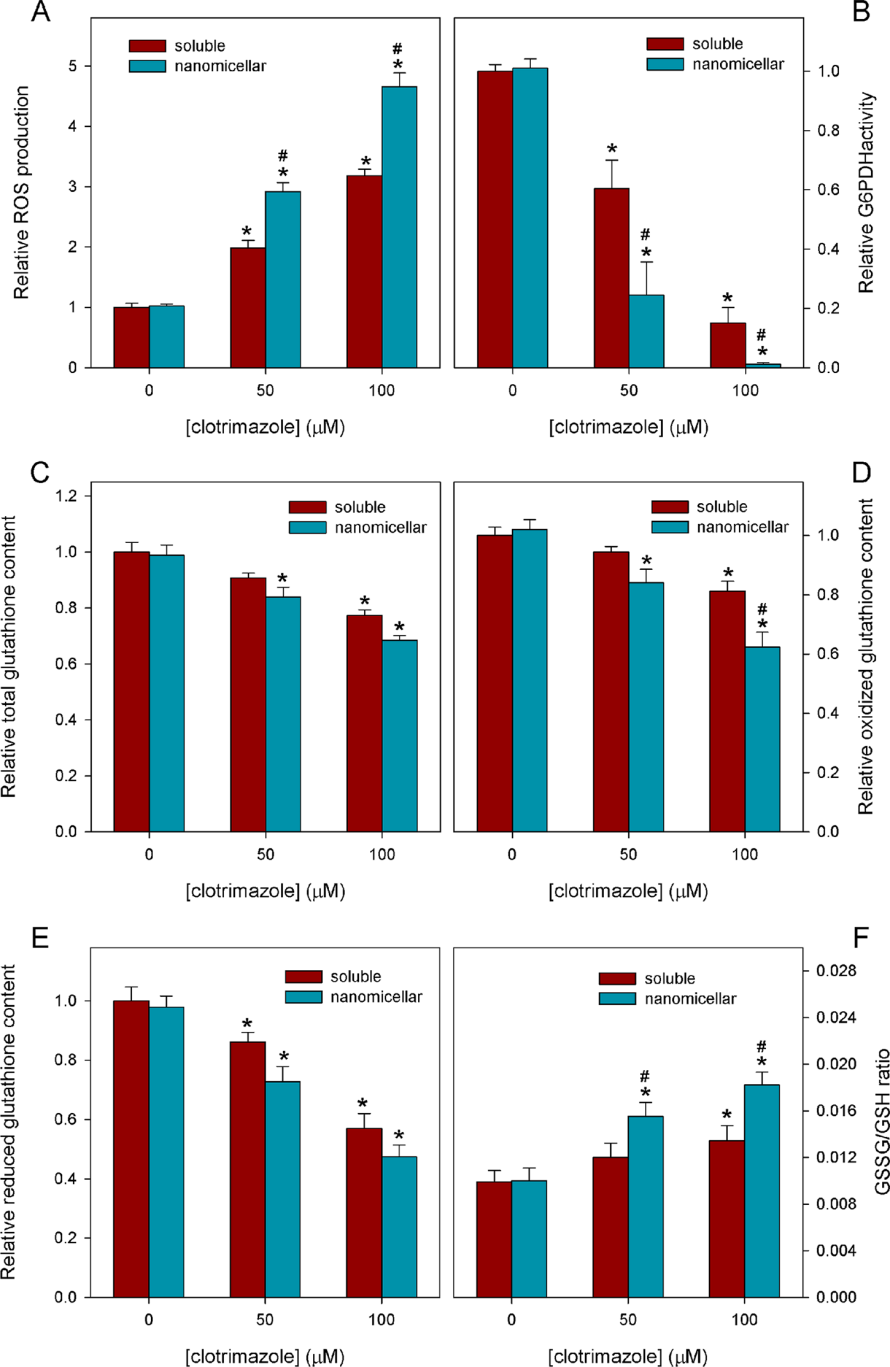
Effects of nanomicellar CTZ on ROS production and oxidative stress protection of MCF-7 cells. ROS production (panel A), G6PDH activity (panel B), total glutathione content (panel C), GSSG content (panel D), GSH content (panel E) and GSSG/GSH ratio (panel F) were assessed as described in Materials and Methods in the absence and in the presence of 50 or 100 μM of DMSO-solubilized or a nanomicellar preparation of CTZ. In the absence of CTZ the appropriate amount of DMSO (1% vol/vol) or CTZ-free nanomicellar preparation was added as controls. The results for these controls were not different from the control with no addition. Values are the means ± standard error of the mean, for at least 4 independent experiments (n = 4). * P < 0.05 compared to control and # P < 0.05 compared to DMSO-solubilized CTZ (two-tailed ANOVA, Bonferroni’s port-hoc test).

Because glycolytic and mitochondrial metabolism are compromised by CTZ, we decided to evaluate oxidative stress in MCF-7 cells treated with CTZ. Reactive oxygen species (ROS) production was evaluated, and the results revealed that CTZ strongly promotes ROS production in MCF-7 cells (Fig 4A). When cells were treated for 24 h in the presence of 50 or 100 μM _s_CTZ, ROS production increased 2 or 3 times, respectively. However, under the same conditions, 50 μM or 100 μM _n_CTZ induced ROS production 3 and 4.7 times, respectively, which is significantly different from the ROS induction by _s_CTZ (Fig 4A; P < 0.05, two-tailed ANOVA, applying Bonferroni’s post-hoc test). Conversely, the antioxidant capacity of MCF-7 cells was evaluated by glucose-6-phosphate dehydrogenase (G6PDH) activity, an indicator of NADPH production, and the reduced and oxidized glutathione (GSH and GSSG, respectively) levels. G6PDH activity was strongly inhibited by both _s_CTZ and _n_CTZ, and _n_CTZ was more efficient at both concentrations used (Fig 4B). It is important to note that the enzyme is almost totally inhibited at 100 μM. Glutathione metabolism was also affected by CTZ, which diminished total glutathione levels; however, the effects of _s_CTZ and _n_CTZ were not significantly different (Fig 4C). The evaluation of GSSG (Fig 4D) and GSH (Fig 4E) levels revealed that both forms were diminished upon CTZ treatment, and the only difference between _s_CTZ and _n_CTZ was in the GSSG levels; at 100 μM, the levels of this metabolite were lower when cells were treated with _n_CTZ than when they were treated with _s_CTZ (Fig 4D). This result was in spite of the fact that 50 μM _s_CTZ had no effect on GSSG levels compared to the control, and there was no significant difference compared to _n_CTZ treatment (Fig 4D). Nonetheless, the evaluation of the GSSG/GSH ratio revealed that _n_CTZ has a stronger effect than _s_CTZ on the anti-oxidant capacity of MCF-7 cells at both 50 μM and 100 μM (Fig 4F).

**Fig 5:**
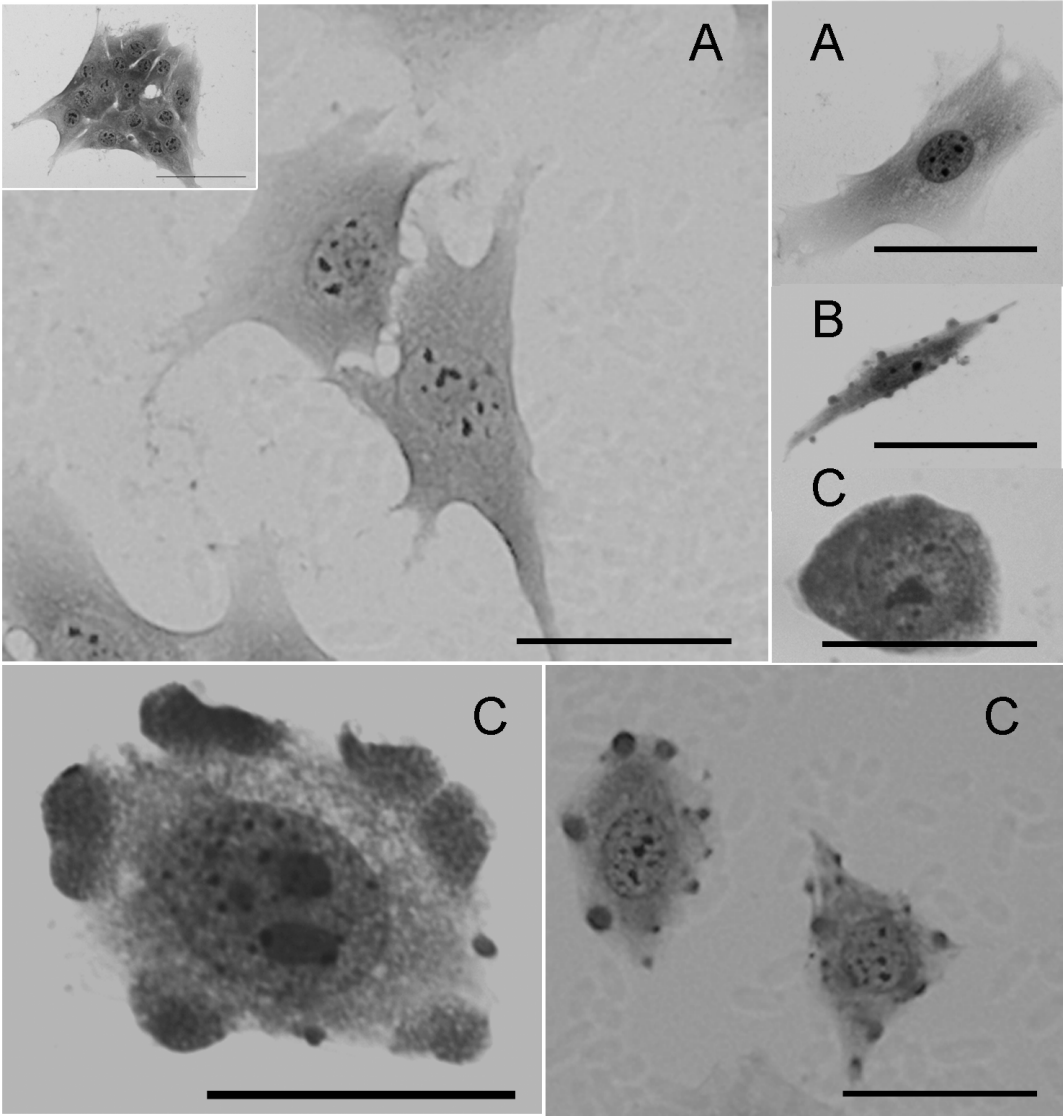
Giemsa optical microscopy of MCF-7 cells treated with nanomicellar CTZ. The experimental procedures are described in Materials and Methods. Panel A: non-treated control cells (inset: MCF-7 cells treated with nanomicelles prepared in the absence of CTZ). Panel B: MCF-7 cells treated with 50 μM _n_CTZ. Panel C: MCF-7 cells treated with 100 μM _n_CTZ. Bar = 50 μm. Images are representative of a series of at least four experiments.

**Fig 6:**
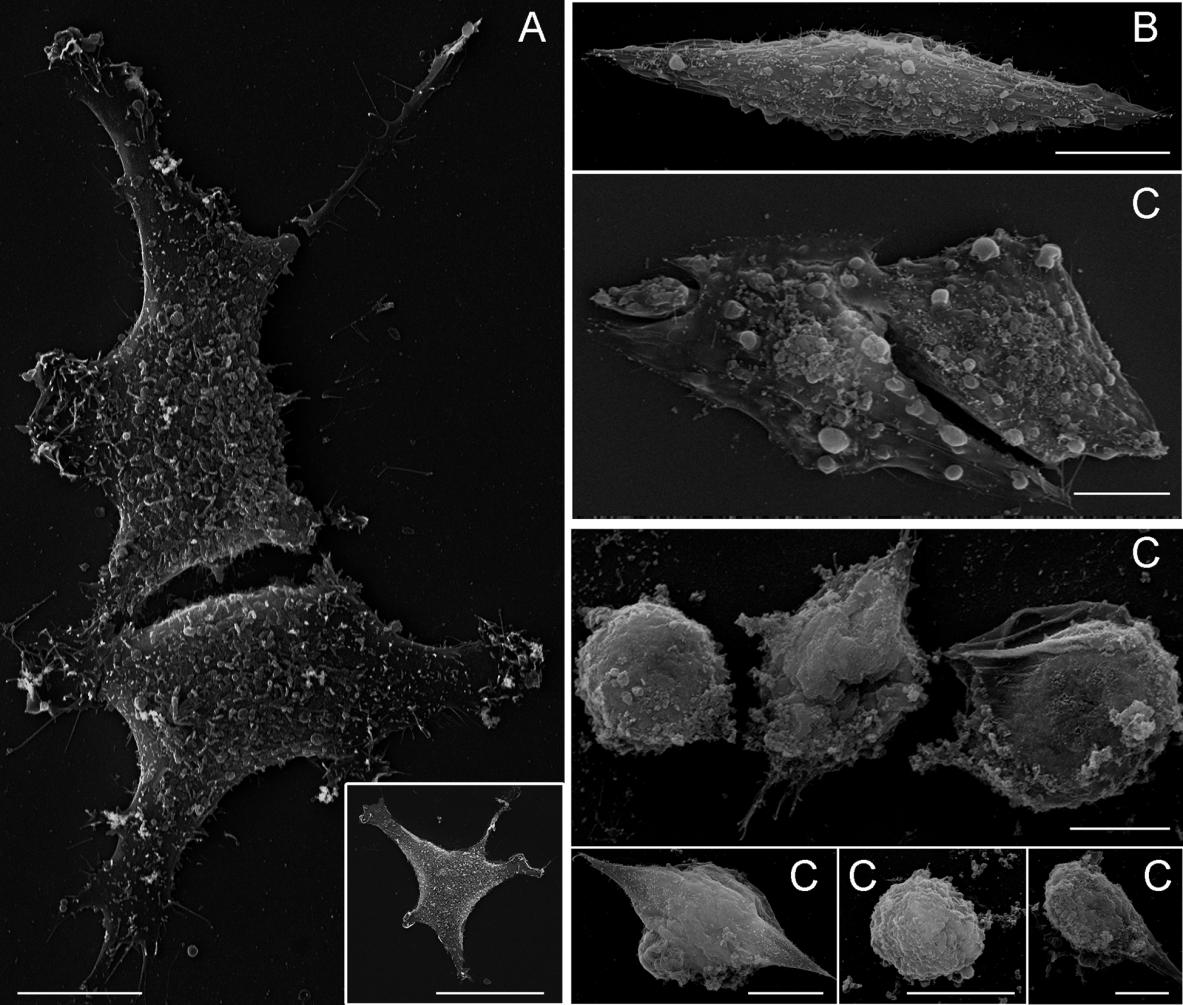
Scanning electron microscopy of MCF-7 cells treated with nanomicellar CTZ. The experimental procedures are described in Materials and Methods. Panel A: non-treated control cells (inset: MCF-7 cells treated with nanomicelles prepared in the absence of CTZ). Panel B: MCF-7 cells treated with 50 μM _n_CTZ. Panel C: MCF-7 cells treated with 100 μM _n_CTZ. Bar = 50 μm. Images are representative of a series of at least four experiments.

_n_CTZ is devastating to MCF-7 cell structure, as revealed by distinct microscopy techniques. Giemsa staining of MCF-7 cells treated for 24 hours with _n_CTZ revealed profoundly affected cell morphology (Fig 5). Compared to control cells (Fig 5, panel A), the treatment of MCF-7 cells with 50 μM _n_CTZ (Fig 5, panel B) converted the stellar-shaped MCF-7 cells into an elongated fusiform morphology lacking protrusions. Moreover, after treatment with 100 μM _n_CTZ, MFC-7 cells become more spherical/elliptical, resembling primitive undifferentiated cells (Fig 5, panel C). The inset to the major panel A of Fig 5 shows MCF-7 cells treated for 24 hours with nanomicelles prepared in the absence of CTZ, and it is clear that they have no effect on cell morphology. These results are corroborated by scanning electron microscopy, which reveals the above alterations in more detail (Fig 6). This technique reveals that the plasma membrane of untreated control cells (Fig 6A) is homogeneously rough. This pattern is altered by treatment with 50 μM _n_CTZ (Fig 6B); the roughness becomes more irregular, and the plasma membrane presents bubbles on its surface (also observed as dense regions in Fig 5). These results are enhanced by treatment with 100 μM _n_CTZ (Fig 6C), which, in addition to the cell shape deformation, clearly disrupts the plasma membrane. Detail in panel C reveals that the plasma membrane integrity is damaged, with membrane fragments around the cells. The lack of effect with empty nanomicelles was also demonstrated by scanning electron microscopy in the inset of Fig 6A.

**Fig 7:**
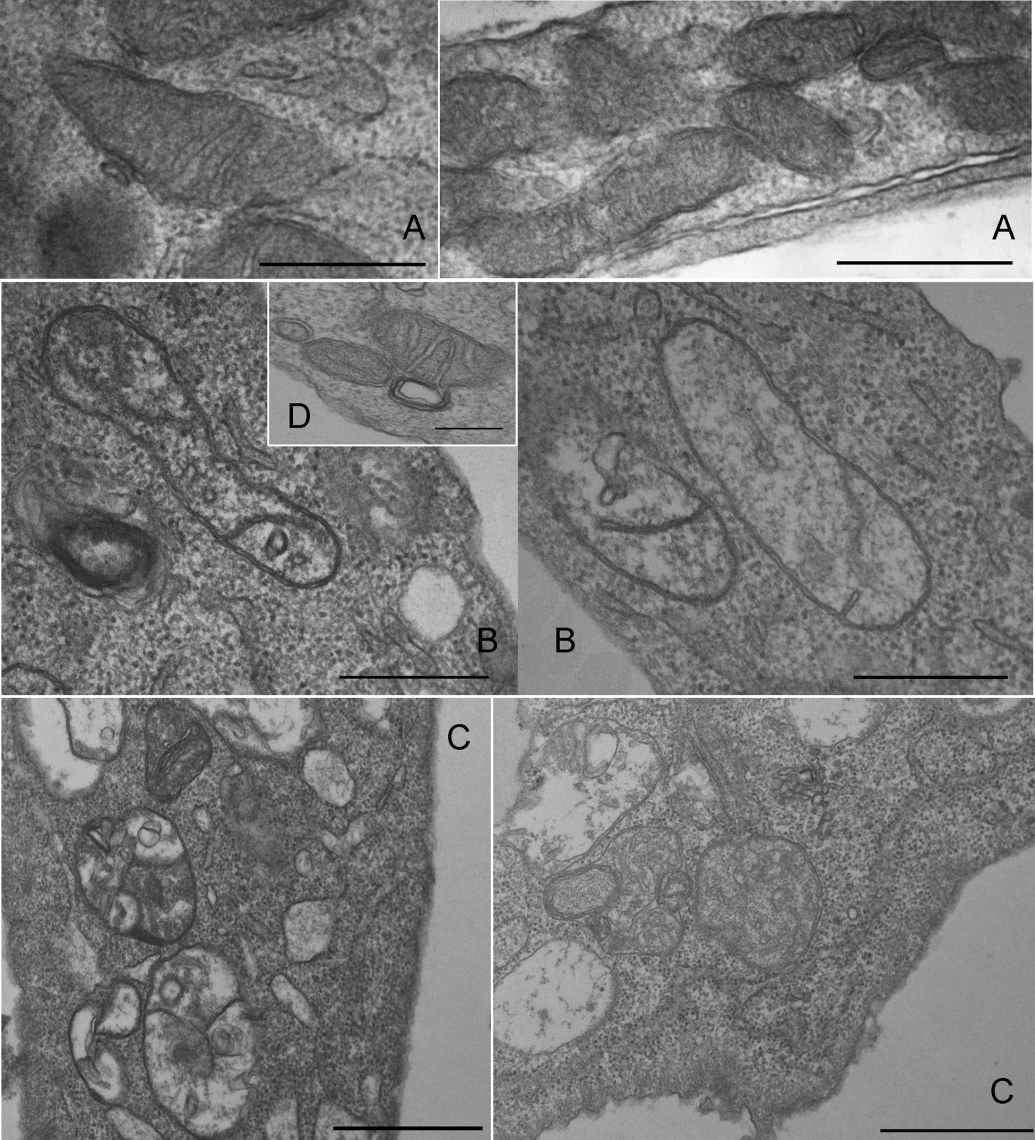
Transmission electron microscopy of mitochondria of MCF-7 cells treated with nanomicellar CTZ. The experimental procedures are described in Materials and Methods. Panel A: non-treated control cells. Panel B: MCF-7 cells treated with 50 μM _n_CTZ. Panel C: MCF-7 cells treated with 100 μM _n_CTZ. Panel D: MCF-7 cells treated with nanomicelles prepared in the absence of CTZ. Bar = 5 μm. Images are representative of a series of at least four experiments.

**Fig 8:**
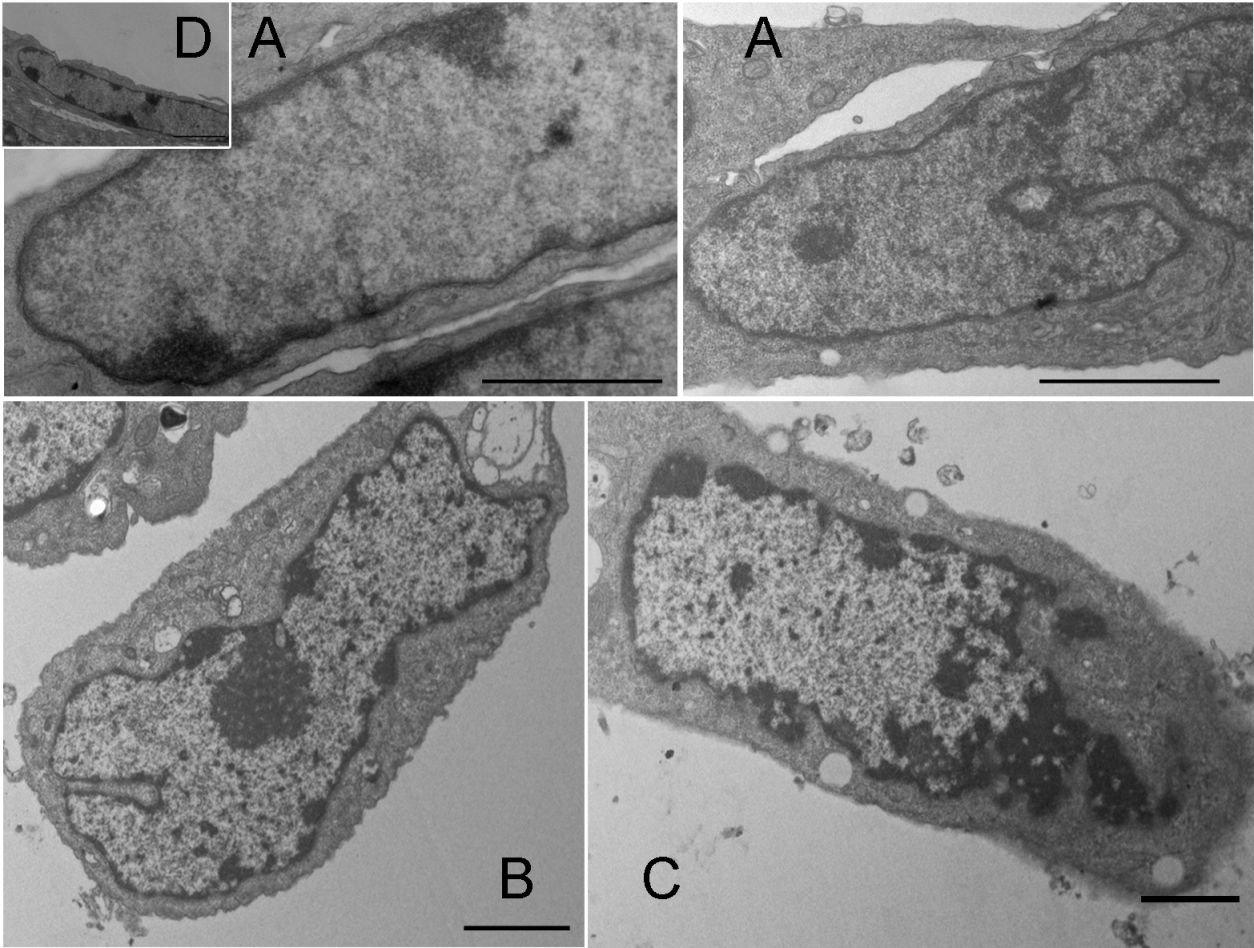
Transmission electron microscopy of the nuclei of MCF-7 cells treated with nanomicellar CTZ. The experimental procedures are described in Materials and Methods. Panel A: non-treated control cells. Panel B: MCF-7 cells treated with 50 μM _n_CTZ. Panel C: MCF-7 cells treated with 100 μM _n_CTZ. Panel D: MCF-7 cells treated with nanomicelles prepared in the absence of CTZ. Bar = 5 μm. Images are representative of a series of at least four experiments.

Mitochondrial structure was also affected by _n_CTZ, as revealed by transmission electron microscopy (Fig 7). Control cells (Fig 7A) and the cells treated with empty nanomicelles (Fig 7D) show a normal mitochondrial profile with a double membrane and parallel cristae. Upon treatment with 50 μM _n_CTZ (Fig 7B), less and shorter cristae structures, a more poorly defined intermembrane space, and a less dense matrix—indicative of the loss of matrix content—were observed compared to the control. These malformations were worsened upon treatment with 100 μM _n_CTZ (Fig 7C); mitochondria became less elongated, cristae almost disappeared, and the matrix was even less dense. The nuclear structure was also affected by _n_CTZ; nuclear condensation was promoted as a function of the drug concentration (Fig 8). It can be observed that upon treatment with 50 μM _n_CTZ (Fig 8B), nuclear condensation is augmented compared to control cells (Fig 8A) and cells treated with empty nanomicelles (Fig 8D), and this effect increases with 100 μM _n_CTZ (Fig 8C).

**Fig 9:**
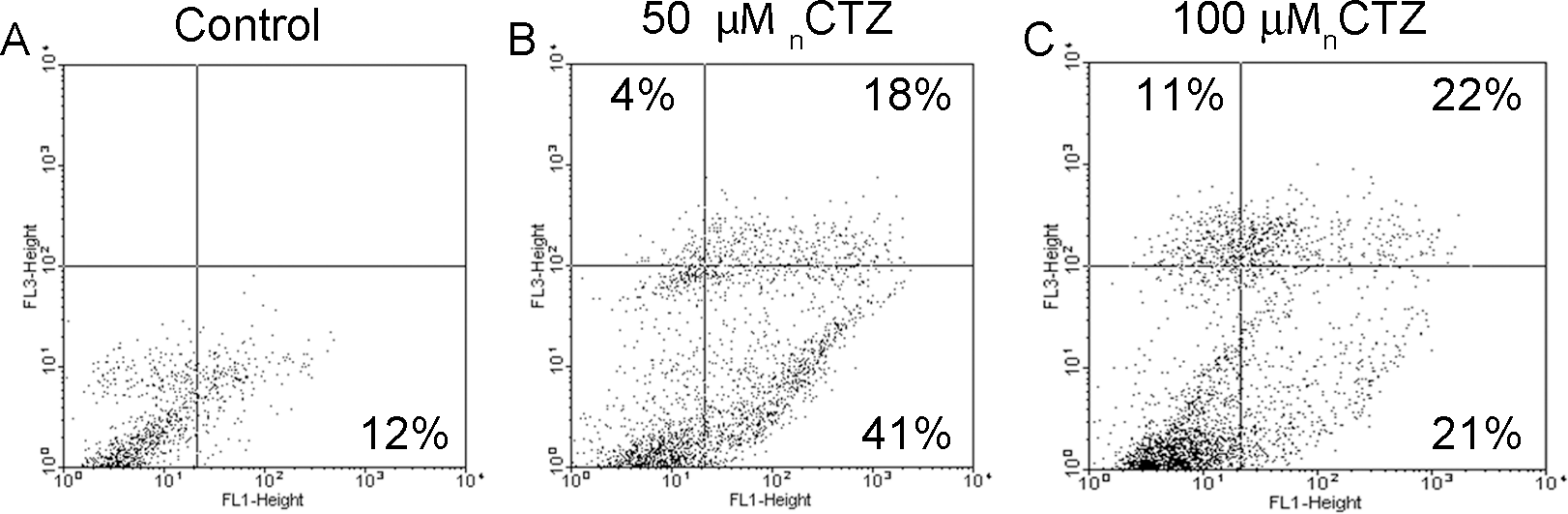
FACS analyses of MCF-7 cells treated with nanomicellar CTZ. The experimental procedures are described in Materials and Methods. Panel A: non-treated control cells. Panel B: MCF-7 cells treated with 50 μM _n_CTZ. Panel C: MCF-7 cells treated with 100 μM _n_CTZ. The percentage of cells in each quadrant is given.

Membrane cell damage and nuclear condensation suggest that both necrosis and apoptosis are occurring as a result of _n_CTZ treatment. Therefore, we evaluated the PI and annexin V staining of MCF-7 cells treated for 24 hours with 50 μM or 100 μM _n_CTZ by FACS analysis. These experiments revealed that the annexin V staining of MCF-7 cells treated with 50 μM _n_CTZ was increased 3.4 times compared to control (Fig 9A and 9B for control and 50 μM _n_CTZ, respectively). Moreover, 50 μM _n_CTZ also promoted necrosis because PI staining was positive after this treatment (Fig 9B). However, after treatment with 100 μM _n_CTZ, significant increases of both PI and annexin V staining were observed (Fig 9C).

## Discussion

Cancer chemotherapy always suffers from the same issue: toxicity. This toxicity can be due to several reasons, such as the lack of tumor specificity of the drug and toxicity to particular organs and tissues. A common example of non-specificity is paclitaxel, which stabilizes microtubules and thus arrests the cell cycle of both normal and tumor cells. Because tumor cells normally cycle faster than non-tumor cells, treatment with paclitaxel is more devastating to tumor cells than to normal cells. Nonetheless, it is undeniable that the whole body is affected by paclitaxel, leading to several undesirable side effects (paclitaxel is used as an example, but the great majority of anti-cancer drugs have similar side effects). We have recently demonstrated that the effects of CTZ on human breast cell proliferation, survival and metabolism are more deleterious to tumor cells than to normal cells [21]. Indeed, the non-tumor human breast cell line MCF-10A was almost unaffected by CTZ for almost all parameters analyzed, including cell proliferation, migration, invasion and metabolism, unlike MCF-7 cells [21]. Moreover, CTZ was more toxic to MDA-MB-231 cells, a more aggressive and invasive breast cancer line, compared to MCF-7 cells. Therefore, we concluded that the more aggressive the cancer cell, the more sensitive it is to CTZ [21]. Furthermore, the selective effects of CTZ on tumors has also been observed in human breast cancer tissue compared to non-tumor fragments obtained from the same patient [22]. Nevertheless, it is undeniable that CTZ as a medicine is very limited due its very low solubility in hydrophilic media, such as blood, and to the fact that the drug inhibits liver P450 complex, which makes it hepatotoxic [43].

The nanoencapsulation of medicinal drugs (nanomedicine) shows promise to improve the efficacy, efficiency and usage of medicines by altering several of their properties. Among these properties, we should highlight drug specificity, tolerability and the therapeutic index. The latter includes the circumvention of hepatotoxicity by avoiding or attenuating the liver metabolism of the nanomedicine [1,37,39]. This property has allowed nanomedicines to be developed that can address pathologies such as cancer, diabetes, HIV infection, malaria, tuberculosis, prion diseases, etc. [39]. Several different materials and techniques have been developed to nanoencapsulate drugs and produce nanomedicine. Most commonly, poly-D,L-lactide-co-glycolide (PLGA), polylactic acid (PLA), poly-ε-caprolactone (PCL), poly-alkyl-cyano-acrylates (PAC), chitosan and gelatin have been used, each with distinct properties and for different applications [39]. The major differences between these materials are the size and stability of the nanoparticles formed, and the choice of the material is thus associated with the efficiency of drug delivery [39]. In this context, nanomicelles have emerged as highly degradable nanoparticles to be designed for the prompt delivery of drugs. Moreover, their structure decreases hydrophobic interactions of the drug with biological systems, thus improving drug delivery [40]. It has been already shown that nanoformulations of CTZ strongly decreases its hepatotoxicity and enhances its bioavailability upon oral administration [31–33].

The current work used nanomicelles of Tween 80 to nanoencapsulate CTZ. Tween 80 has been largely used in pharmaceutical preparations due to its extremely low undesirable effects regardless if administered topically, enterically of parenterically [44]. The formed system presents a nanoscale size (~17-nm diameter) that is not altered by the presence of absence of CTZ. This is a strong indicator that the nanomicelles formed in the presence of CTZ are stable [38]. Indeed, we evaluated the size of these nanomicelles immediately after their preparation and after up to 24 hours at room temperature, and no structural alterations were detected (data not shown). Moreover, our *in vitro* testing clearly demonstrated that these CTZ-containing Tween 80 nanomicelles, here named _n_CTZ, are more toxic than non-encapsulated CTZ to MCF-7 cells. These deleterious effects were evaluated by cell viability assays (MTT assay and leakage of LDH), cell glycolytic capacity (HK, PFK and PK activity, and lactate production rate), mitochondrial potential (SDH activity and transmembrane potential), cellular energy (intracellular ATP content) and the redox state (G6PDH activity, ROS levels, total glutathione, GSSG and GSH).

The three major regulatory glycolytic enzymes, HK, PFK and PK, are strongly inhibited by CTZ, which is in agreement with several reports regarding the effects of this drug on glycolytic enzymes and the whole glycolytic pathway [21–25,35,45–48]. Among these enzymes, PFK is the most sensitive to CTZ and is strongly inhibited by all concentrations tested, further supporting other studies [24,25,48]. Likely due to this strong inhibition, there was no difference between the effects of _s_CTZ and _n_CTZ on this enzyme. In contrast, the effects of _n_CTZ on HK and PK were more pronounced than the effects of _s_CTZ, suggesting that the delivery of CTZ to the cells as a nanomedicine is more efficient than the non-encapsulated drug. We have previously concluded that regardless of whether the inhibition of glycolytic enzymes by CTZ is due to the direct binding of the drug to the enzyme, it is necessary that the drug enter the cell to exert its effects, *i*.*e*., its effects are not mediated by a transmembrane transduction effect [21]. Therefore, it is logical to propose that this entry is somehow facilitated by the nanomicellar preparation of the drug. This result would be easily explained if CTZ were taken up by cells through a carrier-mediated system, such as those proposed for many drugs [49,50]. This facilitated transmembrane drug transport would be more efficient for more media-soluble drugs [49,50]. Because CTZ is very hydrophobic, its inclusion in a water-soluble nanomicellar system could facilitate its interaction with this putative carrier and thereby facilitate its uptake by the cells. The fact is that not only the glycolytic enzymes but also other intracellular and intramitochondrial enzymes, such as G6PDH and SDH, are more affected by _n_CTZ than by _s_CTZ. Moreover, markers of cytosolic and mitochondrial metabolism, such as lactate production, intracellular ATP content, ROS production, GSSG and GSH are also perturbed to a greater extent by _n_CTZ than by _s_CTZ. In the particular case of intracellular ATP, the effects of _n_CTZ are more similar to the effects on SDH than to those observed for the glycolytic enzymes and lactate production (compare Figs 2 and 3). This conclusion is based on the observation that _n_CTZ is only more effective than _s_CTZ at 100 μM for the ATP measurement. This result could lead to the conclusion that, in our experimental model, mitochondrial metabolism is the major ATP-generating pathway or that the effects of CTZ on cellular energy production are more pronounced in mitochondria than in the cytosol. Indeed, CTZ has much less effect on lactate production than it does on the regulatory glycolytic enzymes. The strongest inhibition of G6PDH and the inhibition of PK remove the ability of the pentose phosphate shunt to bypass the initial steps of glycolysis and mostly bypass PFK to support lactate production. Thus, the only explanation for this observation is that once these enzymes are uncontrollably activated in MCF-7 cells [21,46,51–53], their inhibition, although strong, has a minor impact on the total glycolysis rate. This fact is in agreement with the stably activated Warburg effect in these cells [5,12,26,28,29,54,55].

The effects of _n_CTZ are catastrophic to MCF-7 cellular structures, such as the plasma membrane, mitochondria and the nucleus. Moreover, it is clear that _n_CTZ has profound effects on cell shape, which indicates that it alters the cytoskeleton. The interference of CTZ with actin filaments and their associated proteins, as well as with microtubules, has been reported elsewhere [24,25,35,46,48,56]. The destabilization of the cytoskeleton damages the plasma membrane [57], mitochondria [58] and the nucleus [59]. Moreover, the effects of _n_CTZ on these cell structures are certainly aggravated by the increased production of ROS and the attenuation of the cellular antioxidant defense. In particular, the GSSG/GSH ratio is a strong indicator that the _n_CTZ treatment of MCF-7 cells deprives them of a major antioxidant mechanism and thus makes them more susceptible to the deleterious effects of ROS. The relevance of the high GSSG/GSH ratio is reinforced by the extremely low activity of G6PDH upon _n_CTZ treatment because this enzyme is the major source of NADPH (the electron donor that reduces GSSG to re-form GSH). Altogether, these effects promote cell death by inducing apoptosis and then necrosis. It seems that apoptosis is initially triggered, but perhaps due to the devastating force of the treatment, necrosis finally occurs.

### Conclusion

In conclusion, we believe that _n_CTZ may be an alternative vehicle for the systemic administration of CTZ since nanoformulation enhances the drug action and allows its solubility in aqueous media, as demonstrated here, as well as decreases its hepatotoxicity as previously demonstrated [31–33]. Nevertheless, it is clear that nanoformulation strengthens CTZ as an anticancer medicine, and due to the necrosis induction, it should be used at lower doses.

## Materials and Methods

### Materials

Clotrimazole, NAD^+^, NADH, NADP^+^, ATP, ADP, fructose 6-phosphate, lactate, glucose 6-phosphate, phosphoenolpyruvate, hexokinase, lactate dehydrogenase, aldolase, glucose 6-phosphate dehydrogenase, triosephosphate isomerase, and α-glycerophosphate were purchased from Sigma Chemical, St. Louis, MO, USA. Other reagents were of the highest purity available.

### Cell and culture conditions

Human breast cancer MCF-7 cells were obtained from the Cell Bank of the Hospital Universitário Clementino Fraga Filho, UFRJ, Brazil, cultured in DMEM (Dulbecco´s modified Eagle´s medium; Invitrogen, São Paulo, SP, Brazil) supplemented with 10% (v/v) FBS (Fetal bovine serum; Invitrogen) and L-glutamine, and grown at 3700B0030C in 5% CO_2_ as described previously [51].

### Cellular redox state

To assess the cellular oxidative environment, we used DCFH-DA because it reacts with a variety of radical and nonradical species, including hydroxyl and nitroxyl radicals, peroxynitrite, superoxide anion, and hydrogen peroxide. Cells were seeded in 96-well black plates and grown to confluence. The medium was then removed, fresh medium was added, and the cells were incubated with 50 μM DCFH-DA for 30 min before being treated with different concentrations of clotrimazole (0, 50 and 100 μM) for 24 h. We used 10 μM antimycin A as a control to produce superoxide anions. Fluorescence was detected at 485/530 nm excitation/emission using a VICTOR3 multilabel reader (PerkinElmer, Waltham, MA, USA) [60].

### MTT assay

MCF-7 cell viability was assayed by the MTT assay as described previously [53]. Cells were seeded in 96-well plates and grown to confluence. Then, the medium was removed, fresh medium was added, and the cells were returned to the incubator in the presence of different concentrations of clotrimazole (0–100 μM). After 24 h, cells were incubated with 5 mg/mL MTT reagent (3,4,5-dimethiazol-2,5-diphenyltetrazolium bromide, Sigma—Aldrich Co., St. Louis, MO, USA) for 3 h. Thereafter, the formazan crystals were dissolved in DMSO, and the absorbance at 560 nm was evaluated in a VICTOR3 multilabel reader (PerkinElmer, Waltham, MA, USA) with the subtraction of background absorbance at 670 nm [53].

### Lactate assay

Cells were seeded in 96-well plates and grown to confluence. Then, the medium was removed, fresh medium was added, and the cells were treated with different concentrations of clotrimazole (0, 50 and 100 μM) for 24 h. After this incubation, the medium was used to measure extracellular lactate using the EnzyChrom L-Lactate Assay Kit, (BioAssay Systems, Hayward, CA, USA). This system is based on the lactate dehydrogenase-catalyzed oxidation of lactate, which forms NADH that can then reduce a formazan (MTT) reagent. The intensity of product color, measured at 565 nm, is proportional to the lactate concentration.

### ATP quantitative measurement

Cells were seeded in 96-well plates and grown to confluence. Then, the medium was removed, fresh medium was added, and the cells were treated with different concentrations of clotrimazole (0–100 μM) for 24 h. After this incubation, the medium was removed, and the ATP Lite kit reagents (Luminescence ATP Detection Assay System—PerkinElmer, Waltham, MA, USA) were added. This system is based on the production of light by luciferase as it consumes ATP and D-luciferin. The luminescence is proportional to the concentration of cellular ATP and was analyzed with a VICTOR3 multilabel reader (PerkinElmer, Waltham, MA, USA) [21].

### Cell viability

MCF-7, MCF10-A and C2C12 cells were seeded in 96-well plates and grown to confluence. Then, the medium was removed, fresh medium was added, and the cells were returned to the incubator in the presence of different concentrations of clotrimazole (0–100 μM). The micelle components, DMSO and Tween 80 were used as negative controls. After 24 h, the medium was removed, and the amount of leaked lactate dehydrogenase (LDH) was evaluated by monitoring the reduction of NAD^+^ to NADH via the absorbance at 340 nm in a VICTOR3 multilabel reader (PerkinElmer, Waltham, MA, USA) [21].

### Succinate dehydrogenase activity

Cells were seeded in 96-well plates and grown to confluence. Then, the medium was removed, fresh medium was added, and the cells were treated with different concentrations of clotrimazole (0, 50 and 100 μM) for 24 h. After this incubation, the medium was removed, and the cells were snap-frozen at -80°C for 1 h and then incubated for 10 min with 12.3 mM malonate (Sigma-Aldrich, St. Louis, MO, USA), a competitive inhibitor of succinate dehydrogenase (SDH), and 10 mM potassium phosphate buffer (pH 7.6). The cells were then incubated in the dark at 25°C in a reaction buffer containing 12.3 mM diethyl succinate, 0.2 mM 1-methoxy 5-methylphenazinium methyl sulfate, 1.2 mM nitro-blue-tetrazolium (NBT) and 50 mM Tris HCl, pH 7.6. The activity of SDH was determined spectrophotometrically using NBT, which turns purple when it accepts electrons, as an artificial electron acceptor and succinate as the substrate. The purple color is directly proportional to enzyme activity and was measured in a VICTOR3 multilabel reader (PerkinElmer, Waltham, MA, USA) [61].

### Analysis by scanning electron microscopy

Cells were seeded in 24-well plates with glass coverslips on the bottom and grown to confluence. Then, the medium was removed, fresh medium was added, and the cells were treated with different concentrations of clotrimazole (0, 50 and 100 μM) for 24 h. The micelle components, DMSO and Tween 80 were used as negative controls. After this incubation, the coverslips were removed and their adherent cells were washed twice with PBS and fixed in a 0.1 M cacodylate-NaOH buffer (pH 7.2) containing 2.5% glutaraldehyde, 5 mM CaCl_2_ and 3.7% sucrose for 1 h. After that, cells were further fixed for additional 1 h in a 0.1 M cacodylate-NaOH buffer (pH 7.2) containing 1% OsO_4_, 0.8% K_4_Fe(CN)_6_ and 5 mM CaCl_2_. Then, the cells were washed in 0.1 M cacodylate-NaOH buffer (pH 7.2), dehydrated in graded ethanol, and dried with CO_2_ stream. Dried samples were further adhered to 20 nm gold layer-coated scanning electron microscopy stubs using a sputtering device. JEOL JSM 5310 scanning electron microscope (Tokyo, Japan) operating at 25 kV was used to observe the cells.

### Ultrastructural analysis by transmission electron microscopy

Cells were seeded in 24-well plates with glass coverslips on the bottom and grown to confluence. Then, the medium was removed, fresh medium was added, and the cells were treated with different concentrations of clotrimazole (0, 50 and 100 μM) for 24 h. The micelle components, DMSO and Tween 80 were used as negative controls. After this incubation, the coverslips were removed and their adherent cells were washed twice with PBS and fixed in a 0.1 M cacodylate-NaOH buffer (pH 7.2) containing 2.5% glutaraldehyde, 5 mM CaCl_2_ and 3.7% sucrose for 1 h. After that, cells were further fixed for additional 1 h in a 0.1 M cacodylate-NaOH buffer (pH 7.2) containing 1% OsO_4_, 0.8% K_4_Fe(CN)_6_ and 5 mM CaCl_2_. Then, the cells were washed in 0.1 M cacodylate-NaOH buffer (pH 7.2), dehydrated in graded acetone and then embedded in PolyBed 812 (Polysciences Inc., Warrington, PA, USA). Leica (Nussloch, Germany) ultramicrotome was used to obtain ultrathin sections of the dehydrated cells that were stained with uranyl acetate and lead citrate and observed using a FEI MorgagniF268 (Eindhoven, The Netherlands) transmission electron microscope operated at 80 kV.

### Giemsa-stained optical microscopy

Cells were seeded in 24-well plates with glass coverslips on the bottom and grown to confluence. Then, the medium was removed, fresh medium was added, and the cells were treated with different concentrations of clotrimazole (0, 50 and 100 μM) for 24 h. The micelle components, DMSO and Tween 80 were used as negative controls. After this incubation, coverslips were collected daily, rinsed in PBS, fixed in Bouin’s solution, stained with Giemsa, and mounted onto glass slides with Permount (Fisher Scientific, Pittsburgh, PA, USA). The number of adherent cells and the morphological characteristics of the cells were analyzed using a Zeiss Axiovert light microscope (Zeiss, Göttingen, Germany).

### Measurement of Hexokinase, Phosphofructokinase, Pyruvate Kinase and Glucose 6-phosphate dehydrogenase activities

Cells were seeded in 24-well plates and grown to confluence. Then, the medium was removed, fresh medium was added, and the cells were treated with different concentrations of clotrimazole (0, 50 and 100 μM) for 24 h. After this incubation, the cells were trypsinized, centrifuged (10 min x 5000 rpm), resuspended in 10 mM potassium phosphate buffer (pH 7.4), and counted in a hemacytometer. All protein concentrations were analyzed by the method of Lowry [62].

Hexokinase (HK), Phosphofructokinase (PFK), Pyruvate kinase (PK) and Glucose-6-phosphate dehydrogenase (G6PDH) activities were assayed as described previously [21].

### Mitochondrial membrane potential

Cells were seeded in 12-well plates and grown to confluence. Then, the medium was removed, fresh medium was added, and the cells were treated with different concentrations of clotrimazole (0, 50 and 100 μM) for 24 h. After this incubation, the cells were trypsinized, centrifuged (10 min x 5000 rpm), and resuspended in PBS buffer (1 x 10^6^ cells). The mitochondrial membrane potential was measured with rhodamine 123, which is a cell-permeable, cationic, fluorescent dye. MCF-7 cells were incubated with rhodamine 123 (1 mg/mL) for 15 min in the dark. The samples were analyzed with a FACSCalibur flow cytometer (Becton-Dickinson, Franklin Lakes, NJ, USA) equipped with CellQuest software (Joseph Trotter, Scripps Research Institute, San Diego, CA, USA). A total of 10,000 events were acquired in the region previously established to correspond to the MCF-7 cells, and the fluorescence of rhodamine was captured by the respective filter (FL-1: 530 ± 30 nm). The results were analyzed using the "Windows Multiple Document Interface for Flow Cytometry" (WinMDI) application.

### Apoptosis and necrosis detection by flow cytometry

Cells were seeded in 12-well plates and grown to confluence. Then, the medium was removed, fresh medium was added, and the cells were treated with different concentrations of clotrimazole (0, 50 and 100 μM) for 24 h. After this incubation, the cells were trypsinized, centrifuged (10 min x 5000 rpm), and resuspended in PBS buffer (1 x 10^6^ cells). Cell death by apoptosis or necrosis was quantified by using annexin V conjugates or 1 mg/mL propidium iodide (PI), respectively (Molecular Probes, Invitrogen, São Paulo, SP, Brazil). The conjugate for annexin V was Alexa Fluor 488, and the fluorescence was detected at 495/519 nm excitation/emission, whereas PI fluorescence was detected above 670 nm; all measurements were performed with a FACSCalibur flow cytometer (Becton-Dickinson, Franklin Lakes, NJ, USA) equipped with CellQuest software (Joseph Trotter, Scripps Research Institute, San Diego, CA, USA). A total of 10,000 events were acquired. The results were analyzed using the "Windows Multiple Document Interface for Flow Cytometry" (WinMDI) application. Apoptotic or necrotic cells were expressed as percentages of the total number of cells.

### Glutathione analyses

Glutathione quantification was measured using the Glutathione Assay Kit (GSH, GSSG and total) (BioVision, USA). This kit distinguishes between reduced glutathione (GSH), oxidized glutathione (GSSG) and total glutathione. The fluorescent product was read at 420 nm with excitation at 340 nm in a VICTOR3 multilabel reader (PerkinElmer, Waltham, MA, USA).

### Incorporation of CTZ in Oil-Water nanomicelles

The appropriate amount of CTZ was diluted in DMSO and mixed with an adequate volume of water/Tween 80 solution. The system was gently stirred, and the final CTZ molar concentration was 5 mM.

### Analysis of Microemulsions by DLS

The previously prepared microemulsions were analyzed by dynamic light scattering (DLS) using the DynaPro Nanostar DLS system (Wyatt Technology, Santa Barbara, CA, USA). Microemulsions were first centrifuged to ensure that all CTZ was incorporated. Then, the distribution and the size of nanoparticles were analyzed at temperatures from 25–60°C.

### Statistical analysis

Graphs, statistical analyses and non-linear regressions were prepared using the software SigmaPlot 10.0 integrated with SigmaStat 3.1 packages (Systat, CA, USA). All values were presented as the means ± standard error of the mean (S.E.M). Two-tailed ANOVA and Bonferroni’s post-hoc test were used to compare differences among the experimental data. P values ≤ 0.05 were accepted as statistically significant.
